# Comparison of Microscopy and PCR for Detection of *Giardia Lamblia* and *Entamoeba Histolytica* in Human Stool Specimens in a Resource Limited Setting in Western Kenya

**DOI:** 10.4314/ejhs.v30i6.6

**Published:** 2020-11

**Authors:** James Emisiko, Nathan Shaviya, Clement Shiluli, Nathan Kiboi, Ronald Wamalwa, Bernard Jumba, Jeremiah Zablon, Fidelis Mambo, Mustafa Barasa

**Affiliations:** 1 Medical Laboratory Sciences, Masinde Muliro University of Science and Technology, Kakamega, Kenya; 2 Microbiology, Uzima University, Kisumu, Kenya; 3 Biochemistry, Kenyatta University, Nairobi, Kenya; 4 Biomedical Science and Technology, Maseno University, Maseno, Nyanza, Kenya; 5 Immunology, Kenyatta Nairobi Hospital, Nairobi, Kenya

**Keywords:** G.lamblia, E. histolytica, Specificity, Sensitivity, Microscopy, PCR

## Abstract

**Background:**

Accurate diagnosis of Giardia lamblia and Entamoeba histolytica is important since these intestinal parasites account for a significant proportion of morbidity and mortality globally. Microscopy is the key diagnostic test used for diagnosis of the two parasites. Other tests including rapid diagnostic tests and polymerase chain reaction have been developed to improve the detection of these parasites. Most of these newer tests are not affordable in resource limited settings, hence the over reliance on microscopy. The objective of this study was to determine the reliability of microscopy in a resource limited setting in Western Kenya, a region endemic for the two intestinal parasites.

**Methods:**

Polymerase chain reaction, the gold standard test, was performed on stool samples suspected for G. lamblia and E. histolytica. Microscopy was then performed on the same samples and the two tests compared.

**Results:**

Microscopy was found to be 64.4% sensitive, 86.6% specific for the detection of G. lamblia. Additionally, this test was 64.2% sensitive and 83.6% specific for the diagnosis of E. histolytica. Cohen's kappa values of 0.51 and 0.47 were determined for microscopy for G. lamblia and E. histolytica respectively. McNemar's test revealed a significant difference between the two tests, P<0.001.

**Conclusion:**

This study found microscopy to be a reliable diagnostic test in this resource limited setting.

## Introduction

*Giardia lamblia* (syn. *G. duodenalis*; *G. intestinalis*) is the most prevalent intestinal parasite in developing countries with a prevalence rate of 10–50% (1). Moreover, it has been reported as the main cause of diarrhea in day cares as well as travelers from developing countries (2,3). Equally, *Entamoeba histolytica* is a leading cause of diarrhea in developing countries (4). Infection with *E. histolytica* can cause amebic colitis and liver abscess which are associated with high mortality (5). Altogether, these two parasites account for a significant disease burden in developing countries. The World Health Organization (WHO) classifies *G. lamblia* and *E. histolytica* as neglected tropical diseases (6). Part of the WHO 2020 goals are to ensure that these infections are controlled and eliminated (6,7). In order to achieve the WHO 2020 goals, accurate diagnoses of *G. lamblia* and *E. histolytica* is necessary.

Precise laboratory diagnosis of any disease causing pathogen is essential as false results can potentially have severe consequences such as serving as reservoirs for onward transmission and death (8). Additionally, presenting false results can significantly undermine both clinical confidence and credibility of laboratory results, consequently resulting in wrong prescription that may cause drug wastage (9).

Diagnosis of *G. lamblia* and *E. histolytica* is mainly done through microscopic stool analysis of a wet smear or a stained specimen in resource limited settings. Microscopic examination of stool specimens in saline wet mount is a less sensitive technique even when viewed by an expert microscopist (10). Moreover, this technique is often subjective and is prone to misdiagnosis and has other limitations. For instance, microscopy the one cannot distinguish between cysts and trophozoites within degenerated polymorphonuclear cells (4). Center for Disease Control (CDC) recommends examination of adequate samples within 30 minutes of collection to improve sensitivity of microscopy. Also, adequate training of microscopists is often encouraged (11,12). It is important to note that changes in sample pH as well as prior use of antibiotics before sample collection kills trophozoites decreasing sensitivity of microscopy (13). Due to the limitations of microscopy, other techniques have been developed including serological based techniques like Rapid Diagnostic Tests (RDTs) and molecular based techniques such as polymerase chain reaction (PCR) (14–16).

PCR has been adopted as the gold standard method for the diagnoses of amebiasis and giardiasis (4,17). Though, utilization of PCR in routine diagnosis of *G. lamblia* and *E. histolytica* in resource limited settings is impractical. As such, a combination of serologic tests with microscopy detection offers the best approach to diagnosis of *G. lamblia* and *E. histolytica*. However, in most resource limited settings, microscopy is the only diagnostic test used for detection of most enteric parasites. It is important that continuous monitoring and evaluation of the test is done to ensure reliability of microscopy. Moreover, laboratory personnel have different levels of training, experience and skills. Therefore, it is necessary that they are continuously evaluated by confirming what they report to the gold standard. Hence, this study compared the performance of microscopy to PCR in a resource limited setting in Western Kenya.

## Methods

**Specimens**: Fecal samples collected from individuals referred by physicians to Kakamega County division of vector borne and neglected tropical diseases for testing enteric parasites were used in the study. A total of 338 fecal samples were included in this study, consisting of 157 samples suspected for *G. lamblia* and 181 *E. histolytica*. The samples were processed within 30 minutes of sample collection for microscopy. Patient history was taken to determine whether they were on any antibiotics 2 weeks prior to sample collection.

**Microscopy**: A pea size stool sample was collected in sodium acetate-acetic acid-formalin preservative and, after saline washing, divided in two parts. One part was permanently stained with hematoxylin, while the other was concentrated in formol-ethyl acetate for preparation of an iodine wet mount (18). For the wet mount, a drop of lugols iodine was then added and the specimen covered with a glass coverslip. The sample was then mounted on a microscope stage and observed using x10 power objective and x 40 power objective for trophozoites and cysts. Each slide was read by two microscopists, and in case of a disagreement, a third microscopist was called in to confirm the result.

**DNA extraction**: Total DNA was extracted from each *G. lamblia* and *E. histolytica*-positive sample using the QIAamp® Stool mini kit (Qiagen, Germany) following the manufacturer's instructions. To optimize disruption of the cysts, prior to DNA extraction, the samples were subjected to three cycles of freezing and thawing by the following steps: two cycles alternating incubation in liquid nitrogen for five minutes and thawing in water bath at 70°C for five minutes and concluding with a cycle of freezing in liquid nitrogen for five minutes and thawing at 95°C for five minutes. Considering the possibility of false-negative results, negative samples were also processed for DNA extraction.

**PCR Detection**

**Gardia lamblia analysis**: Molecular diagnosis of Giardia was performed using glutamate dehydrogenase (gdh) gene. The eluted DNA was submitted to a semi-nested procedure for amplification of a 432-bp region from the gdh gene according to Read et al.(19). In each reaction, negative (mix + water) and positive (DNA from axenic G. lamblia trophozoites) controls were added. The PCR products were submitted to 1.5% agarose gel electrophoresis, stained with ethidium bromide, and the gel image was recorded under transilluminator UV light.

**Entamoeba histolytica analysis**: This assay was based on the amplification of the small subunit rRNA gene of E. histolytica. The primary PCR for the detection of Entamoeba genus used forward primer, E-1 (5′-TAA GAT GCA GAG CGA AA-3′) and reverse primer, E-2 (5′-GTA CAA AGG GCA GGG ACG TA-3′). The PCR was performed in a 25 µl reaction containing 2.5 µl of 10× PCR buffer, 2 µl of 1.25 mM dNTPs, 1.5 µl of 25 mM MgCl2, 0.5 µl of 10 pmole of each primer, 0.25 µl of 2.5U of Taq polymerase and 2.5 µl of DNA template. Nuclease free water was added to a final volume of 25 µl. The reaction was carried out with an initial denaturing step at 96°C for 2 minutes, followed by 30 cycles of 92°C for 1 minute (denaturation), 56°C for 1 minute (annealing), 72°C (extension) for 90 seconds and a final extension for 7 minutes at 72°C. Subsequently, the primary PCR products were put through a 2^nd^ round of PCR for Entamoeba species specific characterization. Amplification was carried out using the following primer sets: EH-1 (5′-AAG CAT TGT TTC TAG ATC TGA G-3′) and EH-2 (5′- AAGAGG TCT AAC CGA AAT TAG-3′) to detect E. histolytica (439 bp). The secondary amplification used the same concentration of reagents as the primary reaction except that 2.5 µl of the primary PCR product was used as template instead of genomic DNA. The PCR products were analyzed on a 2% agarose gel and visualized under UV light.

**Data analysis**: The completed laboratory register DSA microscopy and PCR results were examined and the information entered into a database by a single data entry clerk using Ms excel sheet SPSS, version 24.0 (IBM, Chicago, USA). Two-by-two contingency table was generated, and with PCR as the gold standard, true positives, false positives, true negatives, false negative, sensitivity and specificity of the tests were determined. In addition, predictive values and Cohen's kappa were determined.

**Ethical considerations**: Ethical approval was obtained from MMUST Institutional Ethics Review Committee. Institutional approval for the study was obtained from medical authorities of the health facility. Confidentiality and privacy of the study subjects were maintained by use of subject identification codes. All the information obtained was strictly confidential. Data were password protected and only accessed by the principal investigator to ensure confidentiality.

## Results

**Diagnosis of *G. lamblia***: The comparison between microscopy and PCR for diagnosis of *Giardia lamblia* is shown oin Table 1. Of the 157 samples, PCR analysis revealed 44 positive and 113 negative for *G. lamblia*.

**Table 1:** Comparison between direct stool analysis and polymerase chain reaction for *G. lamblia*

Method	No. of samples	No. of positives	Sensitivity (%)	Specificity (%)	PPV(%)	NPV (%)	Cohen's Kappa co-efficient	McNemar test (*P*)
**PCR**	157	44	100	100	100	100		
**Microscopy**	157	29	64.4 (48.9–78.1)	86.6 (78.9–92.3)	65.9(53.5–76.5)	85.8(80.1–90.0)	0.51	**<0.001**

Data present as numbers (n) and percentages (%). Data in parenthesis represents 95% confidence interval. PPV; Positive predictive value. NPV; Negative predictive value. *P*<0.05 is bolded.

**Sensitivity, specificity and predictive values of microscopy**: The validity of microscopy is shown in Table 1. Microscopy revealed a sensitivity value of 64.4% (48.9 -78.1) compared with PCR. Hence, the probability of being tested positive when *G. lamblia* is present using microscopy is 0.644. The microscopy revealed a specificity value of 86.6% (78.9 -92.3) compared with PCR. Hence, the probability of being tested negative when *G. lamblia* is absent using microscopy is 0.866. Comparison of microscopy with PCR reported a positive predictive value of 65.9% (53.5- 76.5). Thus, the probability of the patient having *G. lamblia* when the test is positive is 0.659. Furthermore, this diagnostic test gave a negative predictive value of 85.8% (80.1- 90.0) suggesting that the probability of the patient not having *G. lamblia* when the test is negative is 0.858. A Cohen's kappa value of 0.51 indicated a moderate agreement between PCR and microscopy. However, McNemar test revealed a significant difference between the two tests, *P*<0.001.

**Diagnosis of *E. histolytica***: The comparison between microscopy and PCR for diagnosis of *E. histolytica* is shown in Table 2. Since PCR is considered the gold standard for diagnosis of amebiasis, 181 samples were analysed by both PCR and microscopy. PCR revealed 53 positive and 128 negative for *E. histolytica*.

**Table 2:** Comparison between direct stool analysis and polymerase chain reaction for *E. histolytica*

Method	No. of samples	No. of positives	Sensitivity (%)	Specificity (%)	PPV (%)	NPV (%)	Cohen's Kappa co-efficient	McNemar test (*P*)
**PCR**	181	53	100	100	100	100		
**Microscopy**	181	34	64.2 (49.8–76.9)	83.6 (76.0–89.6)	61.8 (51.1–71.5)	84.9 (79.6–89.1)	0.47	**<0.001**

Data present as numbers (n) and percentages (%). Data in parenthesis represents 95% confidence interval. PPV; Positive predictive value. NPV; Negative predictive value. *P*<0.05 is bolded.

**Sensitivity, specificity and predictive values of PCR for detection of *E. histolytica***: Validity of microscopy for the detection of *E. histolytica* is shown in Table 2. Microscopy revealed a sensitivity value of 64.2% (49.8 -76.9) when compared with PCR. Hence, the probability of being tested positive when *E. histolytica* is present using microscopy is 0.642. Additionally, microscopy revealed a specificity value of 83.6% (76.0–89.6). Thus, the probability of being tested negative when *E. histolytica* is absent using microscopy is 0.836. Comparison of microscopy with PCR was reported a positive predictive value of 61.8% (51.1–71.5). Hence, the probability of the patient having *E. histolytica* when the test is positive is 0.618. Furthermore, this diagnostic test presented a negative predictive value of 84.9% (79.6–89.1) suggesting that the probability of the patient not having *E. histolytica* when the test is negative is 0.849. A Cohen's kappa value of 0.47 indicated a moderate agreement between microscopy and PCR. Moreover, McNemar test value showed a significant difference between the two tests, *P*<0.001).

## Discussion

The performance of microscopy for diagnoses of *G. lamblia* and *E. histolytica* in a resource limited setting is important since treatment is solely dependent on it. The current study established that microscopy was 64.4% and 64.2% sensitive for the detection of *G. lamblia* and *E. histolytica* respectively. Moreover, specificity was determined to be 86.6% and 83.6%. Generally, previous studies have reported mixed values of sensitivity and specificity in the detection of these two parasites using microscopy. For instance, a recent study using microscopy Formal Athyl-Acetate (FEA) concentration method for *G. lamblia* reported sensitivity of 31% and specificity of 100% (20). Other studies have revealed sensitivity *vs* specificity; 73% *vs* 100% and 80% *vs* 96.6% in the detection of *G. lamblia* (21,22). On the other hand, sensitivity of microscopy in the diagnosis of *E. histolytica* seems to be varied with some studies reporting values ranging between 10% and 60% (9,23). Diagnostic tests occasionally show varied sensitivities and specificities depending on the setting especially in endemic and non-endemic sites (24,25). As such, Western Kenya is endemic for *G. lamblia* and *E. histolytica* (26,27).

Cohen's kappa coefficient in this study suggests a moderate agreement between microscopy and PCR implying that microscopy could be relied upon in the diagnosis of *G. lamblia* and *E. histolytica*. Contrastingly, McNemar's test indicates a significant difference between microscopy and PCR. However, accurate diagnosis using microscopy is dependent on a number of factors including the skill of the microscopist. Therefore, the difference between the two tests could be as a result of human factors and not absence or presence of the parasites in a sample.

Overall, the findings from our study seem to suggest microscopy as a reliable diagnostic test for *G. lamblia* and *E. histolytica* in this resource limited setting. However, continuous professional training of microscopists should be emphasized to ensure that reliability of the test is maintained. Additionally, processing of the sample should be done within 30 minutes to ensure that integrity of the trophozoites is maintained. Moreover, care should be taken not to alter the pH of the stool samples.

This study found out that microscopy can be a reliable diagnostic test for detection of *G. lamblia* and *E. histolytica* in a resource limited setting. However, specimen processing and skills of the microscopists should be closely monitored to ensure consistency.
